# A Solution with Ginseng Saponins and Selenium as Vaccine Diluent to Increase Th1/Th2 Immune Responses in Mice

**DOI:** 10.1155/2020/2714257

**Published:** 2020-02-21

**Authors:** Yong Wang, Xuemei Cui, Lijia Yuan, Babar Maqbool, Wei Xu, Shanshan He, Ran Guan, Songhua Hu

**Affiliations:** Department of Veterinary Medicine, College of Animal Sciences, Zhejiang University, 866 Yu Hang Tang Rd, Hangzhou, Zhejiang 310058, China

## Abstract

Pseudorabies is an important infectious disease of swine, and immunization using attenuated pseudorabies virus (aPrV) vaccine is a routine practice to control this disease in swine herds. This study was to evaluate a saline solution containing ginseng stem-leaf saponins (GSLS) and sodium selenite (Se) as a vaccine adjuvant for its enhancement of immune response to aPrV vaccine. The results showed that aPrV vaccine diluted with saline containing GSLS-Se (aP-GSe) induced significantly higher immune responses than that of the vaccine diluted with saline alone (aP-S). The aP-GSe promoted higher production of gB-specific IgG, IgG1, and IgG2a, neutralizing antibody titers, secretion of Th1-type (IFN-*γ*, IL-2, IL-12), and Th2-type (IL-4, IL-6, IL-10) cytokines, and upregulated the T-bet/GATA-3 mRNA expression when compared to aP-S. In addition, cytolytic activity of NK cells, lymphocyte proliferation, and CD4^+^/CD8^+^ ratio was also significantly increased by aP-GSe. More importantly, aP-GSe conferred a much higher resistance of mice to a field virulent pseudorabies virus (fPrV) challenge. As the present study was conducted in mice, further study is required to evaluate the aP-GSe to improve the vaccination against PrV in swine.

## 1. Introduction

Pseudorabies (Pr), also known as Aujeszky's disease, is caused by pseudorabies virus (PrV) and it is a highly contagious infectious disease that primarily affects swine, but is also fatal in dogs with signs similar to rabies. The disease causes huge amount of economic losses to the pig industry [1–4]. An official document of the Chinese government has listed Pr as a priority infectious disease that needs to be controlled and eradicated in swine breeding farms by the end of 2020 [5]. Vaccination of swine with attenuated live or inactivated vaccines is commonly practiced on majority of swine farms [6–8]. Live vaccines have been documented to be more efficacious than inactivated vaccines [5, 9]. In China, an attenuated live pseudorabies virus (aPrV) vaccine has been widely used in the control and eradication programs on swine farms since the 1990s. However, incomplete protection against Pr after vaccination has been reported due to poor immune response to vaccines in recent years [2, 8, 10–12]. Though some novel vaccines have been demonstrated to provide effective protection against the current PrV infections, these candidate vaccines are still under preclinical evaluation [13–15], emphasizing the demand for improvement of classical vaccines.

Saponins, consisting of steroid or triterpene aglycone linked to one or more sugars, are a kind of natural product. Through saponins are hemolytic, reports have demonstrated that ginseng saponins shown no hemolytic activity [16, 17]. We previously demonstrated that the saponins isolated from the stem and leaves of *Panax ginseng* C.A. Meyer (GSLS) significantly enhanced the efficacy of vaccination in animals [18–21]. Live vaccines are usually supplied in form of lyophilized powder and diluted in saline before use. Ni et al. developed a solution containing GSLS and thimerosal (TS), which significantly enhanced the immune response when used to replace saline to dilute an aPrV vaccine [22]. Unfortunately, the use of TS is banned in animal vaccines in the latest edition of Chinese Veterinary Pharmacopoeia due to the toxicity of TS with heavy metal mercury which is classified as a nonessential hazardous element [23]. As a trace element, selenium (Se) is essential for regulation of the immune system in both animals and humans [24]. It has been demonstrated to influence the immune system in response to infectious agents [25]. Mahdavi et al. evaluated that the administration of Se nanoparticles with a conventional hepatitis B (HB) antigen vaccine induced a higher immune response with a Th1 bias [26]. Recently, we found that oral administration of GSLS together with injection of Se significantly improved immune response to aPrV vaccine [27]. For clinical convenient, we designed a solution containing GSLS and Se to dissolve the lyophilized powder of aPrV vaccine before vaccination. The present study was to evaluate the effect of the solution on aPrV vaccine in mice by measuring specific antibody responses, lymphocyte proliferation, cytokine productions by lymphocytes, activity of natural killer (NK) cells, and resistance of vaccinated mice to the challenge of field PrV (fPrV).

## 2. Materials and Methods

### 2.1. Mice and Virus

Female ICR mice (6-8 weeks old) were purchased from Shanghai Laboratory Animal Center Co. Ltd. (Shanghai, China). Mice were kept in cages with corncob bedding in a healthy and controlled environment with stable temperature (24 ± 1°C) and humidity (50 ± 10%). Feed and water were provided *ad libitum*. This study was carried out in accordance with the recommendations of Zhejiang University ethics committee. The protocol was approved by the Zhejiang University Animals Ethics Committee (ZJU20160377). fPrV strain WYC001 was kindly provided from Zhejiang Academy of Agricultural Sciences.

### 2.2. Vaccine Preparation

The attenuated commercial swine pseudorabies vaccine, in the form of lyophilized powder, was purchased from Boehringer Ingelheim (Batch: 1951080B; Vetmedica, Inc., USA). The vaccine was diluted either with saline only or with saline containing 30 *μ*g/ml GSLS (Hongjiu Ginseng Industry Co. Ltd., Jilin, China) and/or 10 *μ*g/ml Se (Jinping Chemical Technology Co. Ltd., Shanghai, China), respectively. All solutions were sterilized by passage through a 0.22 *μ*m filter and the endotoxin level of solutions was below 0.5 EU/ml.

### 2.3. Immunization

Mice (*n* = 6/group) were vaccinated twice by intramuscular (i.m.) injection of an aPrV vaccine (1000 TCID_50_, 0.2 ml) diluted in saline or saline containing 2 *μ*g Se and/or 6 *μ*g GSLS at two weeks interval; the control group received no immunization. After immunization, blood samples were collected for detection of serum-specific IgG, isotypes, and neutralizing antibody titers, and splenocytes were isolated for cell proliferation assay, early immune response assay, determination of splenocyte cytokine production analysis of CD4^+^ and CD8^+^ T cells percentage, and transcription factor expression in splenocytes.

### 2.4. Determination of gB-Specific IgG and Isotypes

The commercial pseudorabies virus gB antibody test kit (IDEXX, USA) was used to evaluate, following the manufacturer's instructions, serum gB-specific IgG levels. By using the same ELISA procedure and instructions except that anti-gB monoclonal antibody conjugate was replaced by HRP-conjugated goat anti-mouse IgG1 or IgG2a (1 : 1000), then OD 450 values were detected by the microplate reader (Thermo Scientific, USA) to analyze IgG1 and IgG2a [22, 28].

### 2.5. Determination of PrV TCID_50_

Porcine kidney 15 (PK-15) cells were cultured in DMEM medium (Hyclone, Logan, USA) supplemented with 10% fetal calf serum (FBS; Hyclone), penicillin (100 IU/ml), and streptomycin (100 *μ*g/ml), and 100 *μ*l cells (1 × 10^5^/ml) were seeded in 96-well plates and incubated at 37°C in 5% CO_2_ atmosphere for 24 h. Then, PrV stock was diluted 10-fold with DMEM containing 2% FBS at eight serial dilutions, and 100 *μ*l/well-diluted PrV was added to PK-15 cells in 96-well plates (each dilution repeated for eight times). After incubation (37°C, 5% CO_2_) for 2 h, supernatant was discarded, and 200 *μ*l/well DMEM containing 2% FBS was added into the 96-well plates. Cells without infection of PrV were treated as the control group. Then, plate was continued to incubate at 37°C in 5% CO_2_ condition, and cytopathic effect (CPE) was observed and recorded for 3-4 days, and TCID_50_ was calculated based on the Reed-Muench method.

### 2.6. Analysis of Neutralizing Antibodies

The test was performed as previously described [29, 30] with some modification. Briefly, serum samples were heat-inactivated at 56°C for 30 min. Twofold serial dilutions of sera (in four duplications) with 100 TCID_50_ PrV were incubated for 1 h at 37°C. The virus-serum mixtures were added to confluent monolayers PK-15 cells in 96-well plates. The plates were incubated for 4-5 days at 37°C in 5% CO_2_ atmosphere, and the CPE were recorded. Neutralizing antibody titers were determined by the Reed-Muench method.

### 2.7. Challenge with fPrV

To observe the effect of aP-GSe on survival rate of mice challenged with fPrV after immunization, mice were randomly divided into 4 groups (*n* = 10/group) and received twice injections of saline with or without GSLS-Se or injections of aP-GSe or aP-S at two weeks apart. In a two-week postbooster immunization, mice were challenged with intraperitoneal injection of fPrV (5 × 10^5^ TCID_50_) and mice behaviors were observed for 10 days.

### 2.8. Splenic Lymphocyte Proliferation Response

The assay was performed as previously described [31]. Briefly, the spleens from different groups of mice were isolated under aseptic conditions. Splenocyte suspensions were obtained in RPMI 1640 medium (Hyclone, Logan, USA) supplemented with 10% fetal bovine serum (FBS; Hyclone), penicillin (100 IU/ml), and streptomycin (100 *μ*g/ml). Then, 100 *μ*l splenocytes (5 × 10^6^ cells/ml) were added into 96-well plates and stimulated by 100 *μ*l Con A (5 *μ*g/ml), LPS (5 *μ*g/ml), inactivated PrV antigen (5 × 10^6^ TCID_50_/ml fPrV were heat-inactivated at 56°C for 30 min in water bath), or RPMI 1640 medium. After incubation (37°C, 5% CO_2_) for 48 h, MTT method was conducted and OD 570 values were measured by the microplate reader. Then, the stimulation index (SI) was determined based on the following formula: OD values of stimulated wells/OD values of unstimulated wells.

### 2.9. Analysis of CD4^+^ and CD8^+^ T Cell Percentage in Splenocytes

Splenocytes (5 × 10^6^ cells/ml) were stained with APC-conjugated anti-mouse CD3e (Clone: 145-2C11), FITC-conjugated anti-mouse CD4 (Clone: RM4-5), and PE- conjugated anti-mouse CD8a (Clone: 53-6.7) (BD Pharmingen) for 30 min in dark. Stained splenocytes were harvested by centrifugation (1500 rpm, 25°C, 8 min). Then, cells were resuspended in PBS and were performed on FACS LSR II flow cytometer (BD Biosciences). FlowJo V10 software was used to analyze the data [32].

### 2.10. Cytokine Assay

Splenocytes were restimulated with inactivated PrV antigen (5 × 10^5^ TCID_50_) at 37°C for 48 h in 5% CO_2_ atmosphere. After centrifugation (1500 rpm, 5 min), the quantitative analysis of IFN-*γ*, IL-2, IL-4, IL-6, IL-10, and IL-12 in supernatants was done by commercial ELISA kits (MultiSciences Biotech, Hangzhou, China), according to the manufacturer's instructions.

### 2.11. Quantitative Real-Time PCR (qRT-PCR)

Splenocytes were placed into a 24-well plate at 1 × 10^7^ cells/well and then restimulated with inactivated PrV antigen (5 × 10^5^ TCID_50_) at 37°C in 5% CO_2_ atmosphere for 15 h. Cells were collected after centrifugation (1500 rpm, 4°C, 5 min) and washed with ice-cold PBS. EASY spin Total RNA Extraction Kit (TaKaRa, Dalinan, China) was used to isolate total RNA. Then, PrimeScript™ RT reagent kit (TaKaRa, Dalinan, China) was used to convert total RNA into cDNA. The primers, synthesized by Sangon Biotech, met the NCBI/Primer-BLAST standards and their sequences are listed in Table 1. Quantitative PCR was performed using SYBR Premix Ex TaqTM II (Tli RNaseH Plus) on ABI7300 (PE Applied Biosystems, USA) and data were determined using comparative C_T_ method (2^-△△C^_T_) [33–35], where C_T_ means the fractional cycle number at which the amount of amplified target reaches a fixed threshold, and formula −△△C_T_ = −(△C_T·Target_ − △C_T·Control_), which the relative levels of the target gene mean the fold change of target gene expressions related to the untreated control [36].

### 2.12. Influence of Different Administration Routes of GSLS-Se on Humoral Immune Response

To observe the influences of different administration routes of GSLS-Se on the gB-specific antibody induced by aPrV vaccine, (A) mice were randomly divided into 5 groups (*n* = 6/group) and received i.m. injections twice at two weeks apart as follows: on the left hind limb, groups 1 and 4 were i.m. injected with aP-S and groups 2 and 3 were injected with aP-GSe; on the right hind limb, group 1 was i.m. injected with saline containing GSLS-Se, groups 2 and 4 were i.m. injected with saline alone, and group three was not injected. Group five was injected saline and served as a control group (Table 2). (B) Mice were divided into 5 groups (*n* = 6/group) and received injections twice at two weeks apart of aP-S (groups 1 to 3) or aP-GSe (group 4). Groups 2 and 3 received either saline or saline containing GSLS-Se, respectively, for 3 days before each vaccination. Group 5 was not immunized and served as a control group (Table 3). Each dose contained 1000 TCID_50_. Blood samples were collected a 2-week postbooster vaccination to determine the antibody levels.

### 2.13. Early Immune Response Assay

Serum IFN-*γ* production and cytotoxicity of NK cells were measured to identify the effect of aP-GSe on the early immune response. Blood samples were collected at twenty-four-hour postprimary vaccination to determinate serum IFN-*γ* levels by commercial ELISA kits (MultiSciences Biotech, Hangzhou, China), and then the spleens were isolated to analyze cytotoxicity of NK cells. The cytotoxicity assay was conducted as previously described with some modification [37, 38]. Briefly, 100 *μ*l target YAC-1 cells (2 × 10^6^/ml) were placed into 96-well plates, firstly. Splenocytes from mice acted as effector cells. According to the effector to target (E : T) cell ratios of 25 : 1 and 50 : 1, it modulated the number of splenocytes to5 × 10^7^and 1 × 10^8^ cells/ml, respectively. Then, effector cells (100 *μ*l) were plated in 96-well plates. After incubation (37°C, 5% CO_2_) for 5 h, MTT method was performed and percentage of cytotoxicity was evaluated using the formula: (OD 570 value for target cells − (OD 570 value for target and effector cells − OD 570 value for effector cells))/OD 570 value for target cells × 100%.

### 2.14. Statistical Analysis

Data analysis was performed with GraphPad Prism 7.0 software (San Diego, CA, USA). Multiple comparisons among groups were determined using analysis of one-way ANOVA with the LSD test or two-way ANOVA with Tukey's multiple comparisons test. Student's *t*-test was conducted for data of neutralizing antibodies, and the log-rank test was performed for data of survival. *p* value *<* 0.05 was considered as statistically significant difference.

## 3. Results

### 3.1. GSLS and Se Work Together to Enhance the Antibody Response to aPrV Vaccine

**Figure 1:**
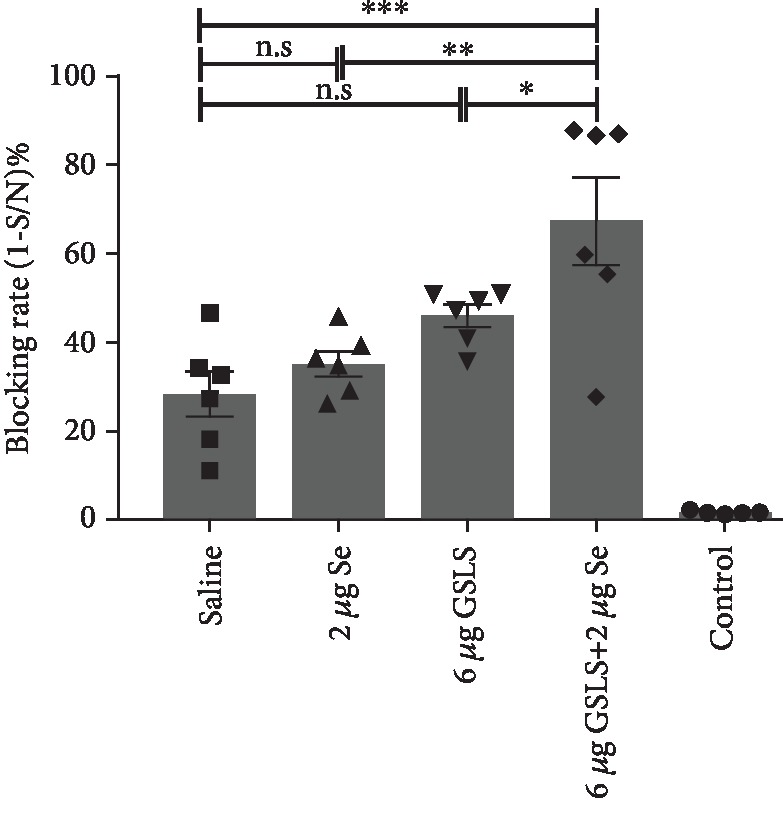
Effects of GSLS and/or Se on PrV gB-specific IgG response. Mice (*n* = 6/group) received twice i.m. injections of an aPrV vaccine (1000 TCID_50_) in saline or saline with Se (2 *μ*g), GSLS (6 *μ*g), or GSLS (6 *μ*g) mixed with Se (2 *μ*g) at two weeks apart. Sera were collected 1 week after the booster for analysis of PrV gB-specific IgG by a blocking ELISA. Data are expressed as the mean ± SE. ^∗^*p* < 0.05, ^∗∗^*p* < 0.01, ^∗∗∗^*p* < 0.001, among the groups as indicated by one-way ANOVA with LSD test.

To observe combined adjuvant effect of GSLS and Se on aPrV vaccine, mice were immunized with aPrV vaccine diluted in saline or saline containing GSLS, Se, or GSLS+Se, and mice injected with saline only served as a control. Data from serum antibody analysis are shown in Figure 1. aPrV vaccine elicited antibody response in all animals. When aPrV vaccine was supplemented with GSLS (6 *μ*g) or Se (2 *μ*g), gB-specific antibody response numerically increased in comparison with aPrV vaccine diluted in saline. When aPrV vaccine was supplemented with GSLS (6 *μ*g) in combination with Se (2 *μ*g), gB-specific antibody response significantly increased when compared with aPrV vaccine in saline. Thus, the combination of GSLS (6 *μ*g) and Se (2 *μ*g) (GSLS-Se) was used as a combined adjuvant in subsequent experiments.

**Figure 2:**
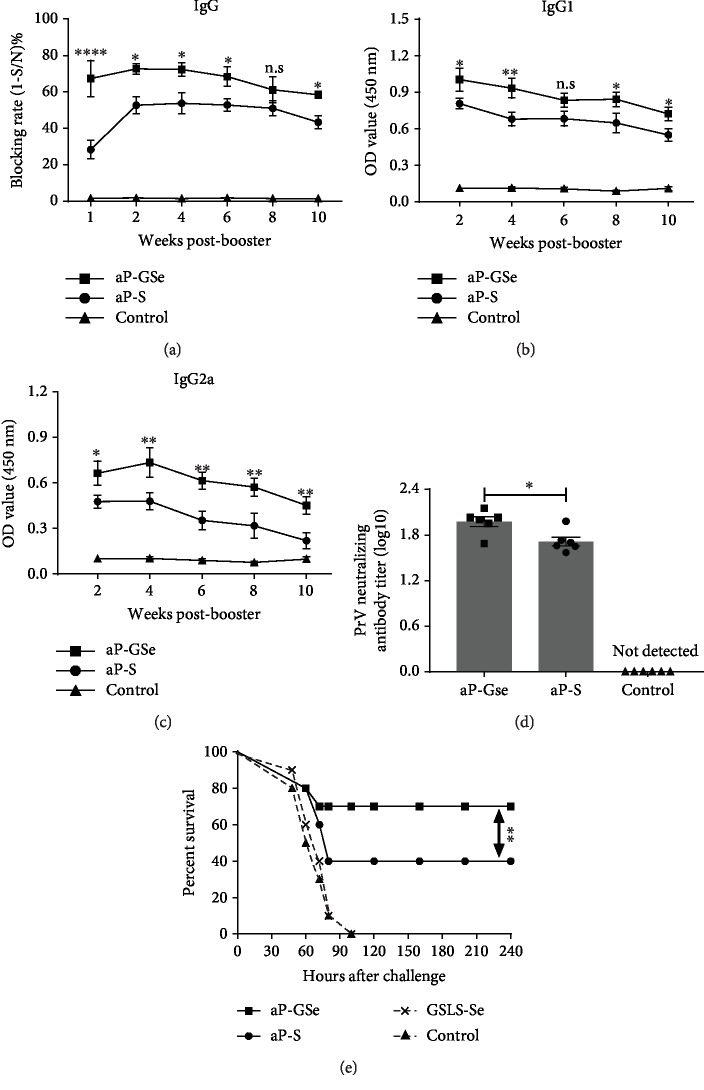
Effect of aP-GSe on antibody responses and survival of mice challenged with fPrV. (1) Mice (*n* = 6/group) received twice i.m. injection of aP-GSe or aP-S at two weeks apart. Mice injected with saline served as control. Sera were collected 1, 2, 4, 6, 8, and 10 weeks after the boost injection for analysis of PrV gB-specific IgG, IgG1, and IgG2a by ELISA (a–c). Serum samples collected 2 weeks after the booster immunization were analyzed for PrV-specific neutralizing antibody titers (d). Data are expressed as the mean ± SE. (2) Mice (*n* = 10/group) received twice i.m. injections of saline or aP-S, GSLS-Se, or aP-GSe at two weeks apart and challenged 2 weeks after boost immunization by intraperitoneal injection of fPrV at a lethal dose of 5 × 10^5^ TCID_50_. The animals were monitored during 240 h and data are expressed as percent survival (e). ^∗^*p* < 0.05, ^∗∗^*p* < 0.01, ^∗∗∗∗^*p* < 0.0001 vs. aP-S group as indicated by two-way ANOVA with Tukey's multiple comparisons test (a–c), Student's *t*-test (d) or log-rank test (e).

To compare the antibody responses induced by aPrV diluted in saline (aP-S) and in GSLS-Se solution (aP-GSe), mice were i.m. administered aP-GSe, aP-S, or saline (control). Serum gB-specific IgG, IgG1, and IgG2a responses during a 10-week postimmunization are shown in Figures 2(a)–2(c). aP-GSe induced significantly higher serum IgG and the isotypes (IgG1 and IgG2a) responses than aP-S alone.

### 3.2. aP-GSe Induces Higher Neutralizing Antibodies

To compare neutralizing antibody titers induced by aP-S and aP-GSe, serum samples collected a 2-week postimmunization were used to estimate the neutralizing antibodies. As shown in Figure 2(d), significantly higher neutralizing antibody titers were induced with aP-GSe than those induced with aP-S.

### 3.3. aP-GSe Enhances Resistance of Animals to fPrV Challenge

To investigate aP-GSe-induced resistance of animals to fPrV challenge, mice were immunized with aP-GSe or aP-S or injected with saline with or without GSLS-Se. In a two-week postimmunization, mice were intraperitoneally challenged with fPrV (5 × 10^5^ TCID_50_) and monitored for 10 days. The results are presented in Figure 2(e). Mice injected with saline or saline containing GSLS-Se began to show convulsions, ataxia, and paralysis at 38 h, and all animals died during 48 to 100 h postchallenge. Of the mice immunized with aP-S, 6 mice began to show neurological signs at 40 h and died during 60 to 80 h postchallenge. Of the mice immunized with aP-GSe, only 3 were found to have neurological signs at 40 h and died during 60 to 72 h postchallenge.

### 3.4. aP-GSe Induces Higher Lymphocyte Proliferation

**Figure 3:**
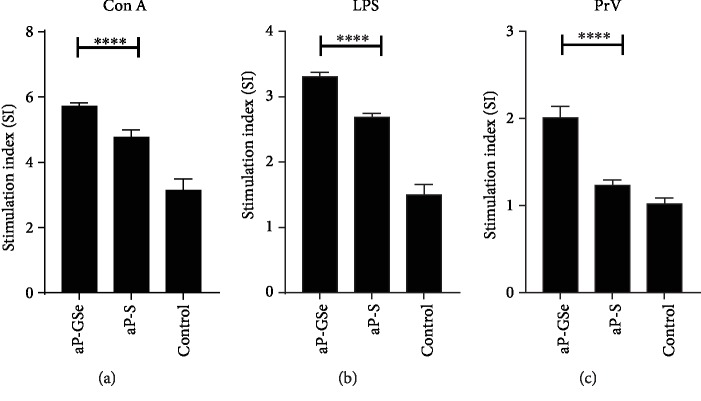
Lymphocyte proliferative response to Con A (a), LPS (b), and PrV antigen (c). Mice (*n* = 6/group) received twice i.m. injections of aP-GSe or aP-S at two weeks apart. Mice injected with saline were served as a control. Splenocytes were prepared 10 weeks after boost immunization and cultured with Con A, LPS, or PrV antigen for 48 h for analysis of lymphocyte proliferation using an MTT method and a stimulation index (SI) was calculated. Data are expressed as the mean ± SE. ^∗∗∗∗^*p* < 0.0001 vs. aP-S group as indicated by one-way ANOVA with the LSD test.

Lymphocyte proliferation is an indicator of the cell-mediated immune response. To compare cellular immune responses induced by aP-GSe and aP-S, splenic lymphocytes were prepared from mice immunized with aP-GSe and aP-S, and lymphocyte proliferative responses to Con A, LPS, and inactivated fPrV antigen were tested.  Figure 3 shows that mice immunized with aP-GSe had significantly higher lymphocyte proliferative responses to Con A, LPS, and fPrV than the mice immunized with aP-S.

### 3.5. aP-GSe Increases Ratio of CD4^+^ and CD8^+^ T Cells

**Figure 4:**
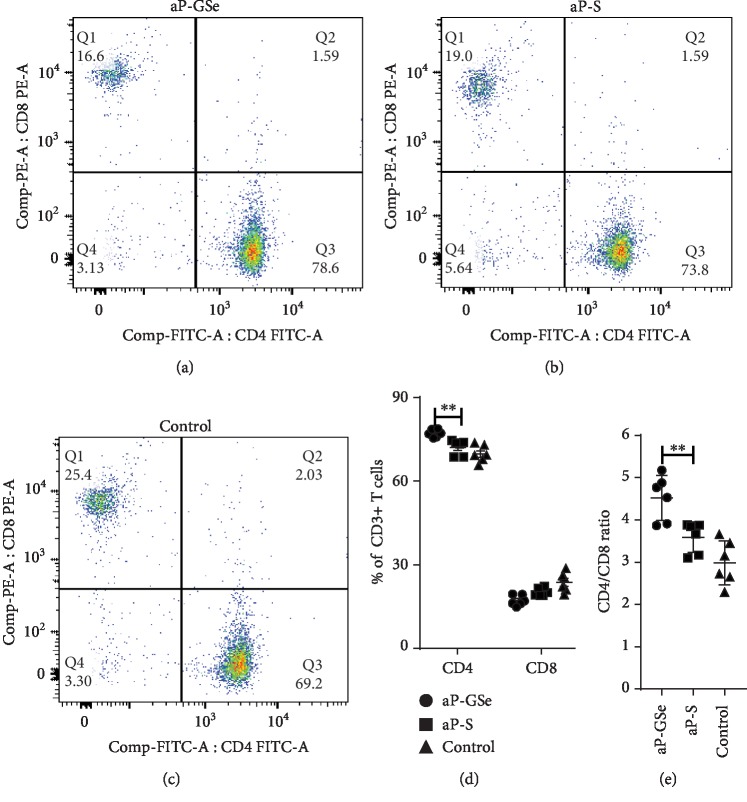
Quantification of splenic CD3^+^CD4^+^ and CD3^+^CD8^+^ T cell subpopulations and the CD3^+^CD4^+^/CD3^+^CD8^+^ratio. Mice (*n* = 6/group) received twice i.m. injection of aP-GSe or aP-S at two weeks apart and animals injected with saline served as a control. Splenocytes were prepared 2 weeks after boost immunization for analysis of CD3^+^CD4^+^ and CD3^+^CD8^+^ T cell subpopulations by flow cytometry. Data are expressed as the mean ± SE. ^∗∗^*p* < 0.01 vs. aP-S group as indicated by one-way ANOVA with the LSD test.

Host immunity is associated with the ratio of CD4^+^ and CD8^+^ T cells. To compare CD4^+^ and CD8^+^ T cells in mice immunized with aP-GSe and aP-S, splenocytes were isolated from mice immunized with aP-GSe and aP-S for flow cytometric analysis. Results showed that aP-GSe significantly increased CD4^+^ T cell percentage (Figures 4(a)–4(d)) and CD4^+^/CD8^+^ ratio (Figure 4(e)) of splenic CD3^+^ T cells when compared with aP-S.

### 3.6. aP-GSe Modulates Cytokine Production

**Figure 5:**
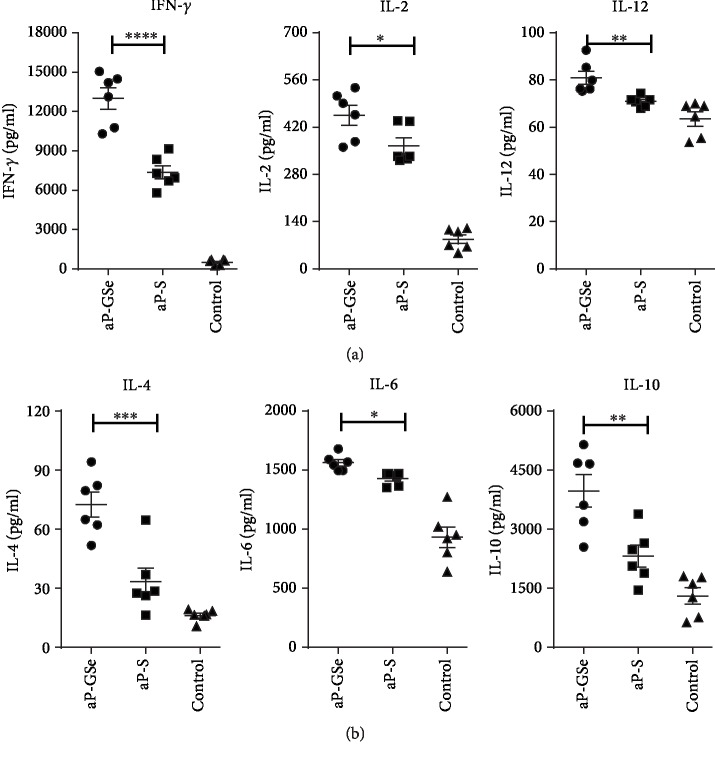
Production of IFN-*γ*, IL-4, IL-2, IL-6, IL-12, and IL-10 by splenocytes. Mice (*n* = 6/group) received twice i.m. injection of aP-GSe or aP-S at two weeks apart. Mice injected with saline served as control. Splenocytes were prepared 2 weeks after boost immunization and cultured with PrV antigen for 48 h. The supernatants were harvested for analysis of (a) Th1-type cytokines (IFN-*γ*, IL-2, IL-12) and (b) Th2-type cytokines (IL-4, IL-6, IL-10) by ELISA. Data are expressed as the mean ± SE. ^∗^*p* < 0.05, ^∗∗^*p* < 0.01, ^∗∗∗^*p* < 0.001, ^∗∗∗∗^*p* < 0.0001 vs. aP-S group as indicated by one-way ANOVA with the LSD test.

To compare cytokines produced by splenocytes isolated from the spleen of mice immunized with aP-GSe and aP-S, cytokine production was measured in splenocytes stimulated with PrV antigen.  Figure 5 shows that mice immunized with aP-GSe had significantly higher production of cytokines IFN-*γ*, IL-4, IL-2, IL-6, IL-12, and IL-10 by splenocytes.

### 3.7. aP-GSe Increases GATA-3 and T-bet mRNA Expression

**Figure 6:**
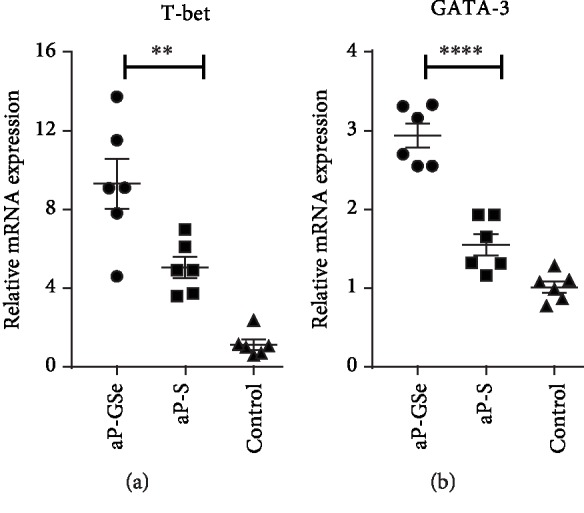
T-bet (a) and GATA-3 (b) mRNA expressed by splenocytes. Mice (*n* = 6/group) received twice i.m. injection of aP-GSe or aP-S at two weeks apart and mice injected with saline served as a control. Splenocytes were prepared 2 weeks after boost immunization and cultured with PrV antigen for 15 h. Fold change of relative mRNA expression of T-bet and GATA-3 was analyzed by RT-qPCR. Data are expressed as the mean ± SE. ^∗∗^*p* < 0.01, ^∗∗∗∗^*p* < 0.0001 vs. aP-S group as indicated by one-way ANOVA with the LSD test.

Transcription factors T-bet and GATA-3 regulate expression of Th1 and Th2 cytokines [39]. To compare transcription factors T-bet and GATA-3 expressed in splenocytes from mice immunized with aP-GSe and aP-S, expression of T-bet and GATA-3 mRNA was analyzed by quantitative real-time PCR in splenocytes stimulated with PrV antigen.  Figure 6 shows mice immunized with aP-GSe had significantly higher expression of T-bet and GATA-3 mRNA than mice immunized with aP-S.

### 3.8. Effect of Different Administration Routes of GSLS-Se on the Antibody Response

**Figure 7:**
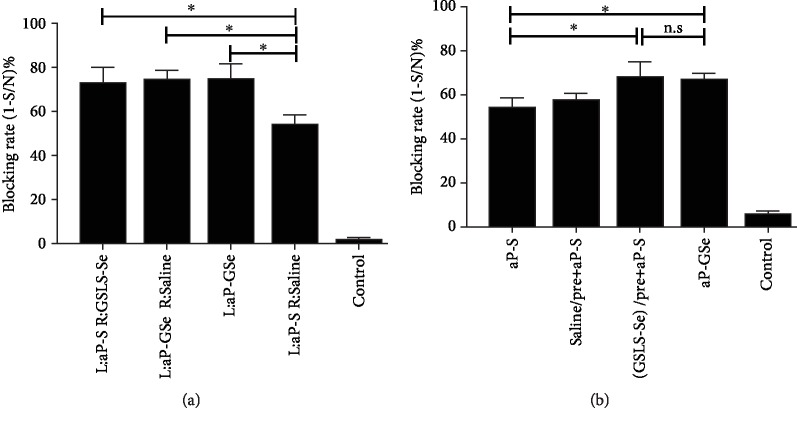
Effect of administration routes of GSLS-Se on IgG response. (a) Mice (*n* = 6/group) were i.m. injected with saline with or without GSLS-Se on their right hind limb (R) before immunization on the left hind limb (L). (b) Mice (*n* = 6/group) were i.m. injected with saline with or without GSLS-Se for 3 days before immunization. Immunization was i.m. administered with aP-GSe or aP-S at two weeks apart. Control animals were not immunized but injected with saline. Sera were collected 2 weeks after the booster for analysis of PrV gB-specific IgG. Data are expressed as the mean ± SE. ^∗^*p* < 0.05, among the groups as indicated by one-way ANOVA with LSD test.

To observe antibody responses influenced by different administration routes, we first injected aPrV vaccine and GSLS-Se together or separately and then injected GSLS-Se before immunization in mice. Results showed that administration of GSLS-Se together or separately with aPrV vaccine (Figure 7(a)) or injection of GSLS-Se before immunization (Figure 7(b)) significantly increased antibody response.

### 3.9. aP-GSe Elicits an Early Immune Response

**Figure 8:**
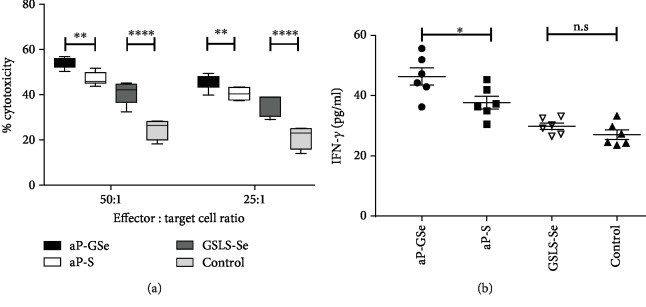
Cytotoxicity of NK cells (a) and IFN-*γ* response (b). Mice (*n* = 6/group) received i.m. injection of aP-GSe or aP-S, or just injection of saline with or without GSLS-Se. In twenty-four-hour postimmunization, splenocytes were isolated for analysis of cytolytic activity to YAC-1 cells by an MTT method, and blood samples were collected for analysis of IFN-*γ* by an ELISA. Data are expressed as the min to max (a) or mean ± SE (b). ^∗^*p* < 0.05, ^∗∗^*p* < 0.01, ^∗∗∗∗^*p* < 0.0001 vs. aP-S group or control as indicated by two-way ANOVA with Tukey's multiple comparisons test (a) or one-way ANOVA with LSD test (b).

To observe the early immune response elicited by aP-GSe, cytotoxic activity and serum IFN-*γ* were evaluated. Results showed that aP-GSe induced significantly higher cytotoxic activity (Figure 8(a)) and serum IFN-*γ* production (Figure 8(b)) than aP-S.

## 4. Discussion

GSLS is an extract made from *Panax ginseng* C.A. Meyer and has been demonstrated to be effective for improving various animal vaccines. Previously, Ni et al. showed that a solution containing GSLS and TS significantly improved immune responses to the aPrV vaccine in mice [22]. However, the U.S. Food and Drug Administration (FDA) has worked with vaccine manufacturers to reduce or eliminate TS from vaccines, and TS in U.S. FDA-licensed vaccines has significantly declined [40, 41]. Also, TS use has been banned recently in animal vaccines in China. Considering that the diluent solution developed by Ni et al. is not only effective on swine (data not shown) but also simple to fabricate, cheaper, and easy to use, we search for a safe alternative diluent for aPrV vaccine. Unlike TS, Se is an essential trace element and has been found to improve both the innate and the adaptive immune responses [42–44]. We tried to replace TS with Se to develop a new formulation for dilution of aPrV vaccine. The present study demonstrated that aPrV vaccine diluted in the new solution containing GSLS and Se (aP-GSe) induced significantly higher gB-specific antibody response than the vaccine diluted in saline (aP-S). As one of the envelope glycoproteins of PrV, gB has been demonstrated to modulate the immune response [3]. Studies show that animals inoculated with purified gB can survive a lethal PrV challenge [45]. Moreover, a plasmid DNA vaccine expressing gB glycoprotein not only induced the strongest humoral and cellular immune responses but also exhibited the highest protection rate against virulent PrV challenge as compared to gC and gD [46, 47]. Therefore, higher gB antibody response to aP-GSe detected in the present study indicated that higher protection was induced by aP-GSe.

Humoral immune response contributes a vital role in the immune defense against PrV [48, 49]. In mice, subclasses of IgG include IgG1, IgG2a, IgG2b, and IgG3. Mostly, the viral infections induce antibody-mediated responses with the predominance of IgG2a in mice [50–52]. Figures 2(a)–2(c) show that aP-GSe induced significantly higher IgG, IgG1, and Ig2a responses during a 10-week postbooster immunization. Enhanced antibody responses were associated with the increased survival rate (70%) of mice vaccinated with aP-GSe while only 40% of mice vaccinated with aP-S in the challenge experiment with lethal dose of fPrV two weeks after the second immunization (Figure 2(e)). This result paralleled the neutralizing antibodies when mice were vaccinated with aP-GSe (Figure 2(d)).

Lymphocyte proliferation is dependent on mitogens used for a test. T cell response is induced by Con A while the B cell response is induced by LPS [53]. In comparison to aP-S, lymphocyte proliferative responses to Con A, LPS, and PrV antigen were significantly improved in the aP-GSe group (Figure 3), suggesting that both T and B cells were activated. Clonal expansion is essential for triggering B lymphocytes to induce the antibody production. The increased lymphocyte proliferative response to PrV antigen was consistent with the enhanced gB-specific IgG and isotypes response in mice which received the aP-GSe (Figures 2(a)–2(c)).

IgG2a, IL-2, TNF-*α*, and IFN-*γ* are related to Th1 immune response which is effective for protection against intracellular infections [54], while the Th2 immune response is required for protective immunity against extracellular infections such as parasitic infections, including those caused by helminths, and characterized by the production of IL-4, IL-5, IL-6, and IL-10 cytokines and IgG1 [55, 56]. Cytokines play a crucial role in differentiation of naive CD4^+^ T cells [57].  Figure 5 shows that aP-GSe enhanced production of not only IFN-*γ*, IL-2, and IL-12 but also IL-4, IL-6, and IL10, suggesting that both Th1 and Th2 subset cells were activated [58–61]. Cytokine-cytokine receptor interaction on naive CD4^+^ T cells activates the Janus kinase and signal transducers and activators of transcription (JAK-STAT) pathways, JAK1/2 and STAT1/3/4 induced by IFN-*γ* and IL-12 to stimulate T-bet and further IFN-*γ* production, and IL-4 triggers JAK1/3 and STAT6 to activate transcription factors GATA-3 [57]. In Figure 6, expressions of transcription factors T-bet and GATA-3 were significantly increased in the aP-GSe group. And T-bet and GATA-3 are believed to regulate naive CD4^+^ T cells to differentiate toward Th1 and Th2 cells [62]. IL-6 is a cytokine with multiple biologic effects that modulates inflammatory and immune responses. Reports demonstrated that activation of the IL-6/JAK2/STAT3 pathway promoted the differentiation of B cells into plasma cells [63, 64]. Besides, IL-10 is also a pleiotropic cytokine exert biological effect via the JAK1 and STAT1/3 pathways [65]. Hence, we proposed that aP-GSe-enhanced Th1 and Th2 immune responses might be attributed to the JAK-STAT pathways underlying.

The ratio of CD4^+^/CD8^+^ is usually used to evaluate the immune status of the body, and higher CD4^+^/CD8^+^ ratio indicates a stronger immune status [66, 67]. Yang et al. found that inoculation with foot-and-mouth disease (FMD) vaccine leads to higher immune response and CD4^+^/CD8^+^ ratio of lymphocytes in young sires [68]. Su et al. reported that the percentage of CD4^+^ T cells and CD4^+^/CD8^+^ ratio was significantly higher in the mice vaccinated with rabies virus vaccine plus ginsenoside Re than the control [69]. Bianchi et al. demonstrated that depletion of CD8^+^ T cells had no influence on protection against lethal PrV challenge while anti-CD4 treatment reduced the protection of animals by 30% after lethal challenge [70]. The present result showed that the percentage of CD4^+^ T cells and ratio of CD4^+^/CD8^+^ was significantly increased when aP-GSe was used in comparison with the aP-S group (Figure 4). Increased percentage of CD4^+^ T cells and ratio of CD4^+^/CD8^+^ paralleled the higher protection induced by aP-GSe (Figure 2(e)).

Adjuvants may exhibit different effects when administration routes are different. For instance, De Gregorio et al. reported that the adjuvant effect was often lost when the antigen and alum were administered at separate locations [71]. Thoryk et al. demonstrated that lipid nanoparticles (LNPs) must be coadministered with antigens to exert adjuvant property [72]. However, Rivera et al. observed that ginseng improved antibody response to porcine parvovirus (PPV) antigen regardless of that the ginseng and antigen were coinjected or not [73]. Similar to the results found by Rivera et al., this study observed that GSLS-Se enhanced the gB-specific antibody response to the aPrV vaccine regardless of it was injected together or separately with vaccine (Figure 7(a)). Li et al. found that oral administration of GSLS prior to immunization significantly enhanced the immune response of mice to FMD vaccine [18]. Saade et al. reported an adjuvant effect of Advax™ (a polysaccharide) when injected 24 h prior to administration of hepatitis B surface antigen, while no adjuvant effect was observed when alum was injected prior to vaccination [74]. We found that injection of GSLS-Se before immunization significantly increased gB antibody response to aPrV vaccine (Figure 7(b)). Based upon these results, we hypothesized that GSLS-Se may be able to enhance the adaptive immune response via stimulating the innate immunity such as NK cells.

Activated NK cells secrete cytokines including IFN-*γ* and have direct cytotoxic effect on virus-infected cells [75]. In the present study, we found that aP-GSe induced significantly higher NK cell activity and IFN-*γ* production (Figure 8), suggesting that injection of aP-GSe activated an early host innate immune response.

The significantly enhanced immune responses observed in the present study may result from the combined immunomodulatory effects of GSLS and Se. According to HPLC analysis, GSLS used in the present study contained Re (16.4%), Rd (9.0%), Rg1 (6.0%), Rb2 (3.8%), Rc (3.7%), Rb1 (2.4%), and Rf (0.1%). Hu et al. reported that coinjection of ginsenoside Rb1 with *Staphylococcus aureus* vaccine induced significantly higher antibody levels in dairy cattle [76]. Song et al. demonstrated that ginsenoside Re significantly improved Th1 as well as Th2 immune responses to an inactivated H3N2 influenza virus vaccine in mice [77]. Su et al. discovered that coadministration of Re with an inactivated rabies virus vaccine significantly improved the Th1 as well as Th2 immune responses in a mouse model [69]. Bi et al. demonstrated that oral administration of Rg1 exhibited adjuvant property to an attenuated live infectious bursal disease vaccine [78]. NF-*κ*B exerts an important function of the transcription control of proteins needed for many innate and adaptive immune responses. Olafsdottir et al. reported that the NF-*κ*B signaling pathway was involved in mechanism of action for some licensed adjuvants [79]. Most importantly, Su et al. found that Re or Rg1 exerted its adjuvant effect to both Th1 and Th2 immune responses via the TLR4/NF-*κ*B signaling pathway [31]. Taken together, we believe that the molecular mechanisms underlying for adjuvant effect of GSLS-Se in the present study may be at least related to the fractions Re, Rg1, and Rb1 contained in GSLS by activation of the NF-*κ*B and JAK-STAT signaling pathways.

In conclusion, the present study demonstrated that aPrV vaccine diluted in saline containing GSLS and Se induced significantly higher immune responses than the vaccine diluted in saline only. aP-GSe elicited significantly higher Th1 (IgG2a, IFN-*γ*, IL-2, IL-12) and Th2 (IgG1, IL-4, IL-6, IL-10) immune responses in association with upregulated T-bet/GATA-3 mRNA expression. aP-GSe also significantly increased cytolytic activity of NK cells and lymphocyte proliferative response to Con A, LPS, and PrV antigen as well as CD4^+^/CD8^+^ ratio when compared to aP-S. More importantly, we observed significantly higher neutralizing antibody titers and protection against fPrV challenge in mice injected with aP-GSe than those mice injected with aP-S. Therefore, saline containing GSLS and Se may be a promising solution used to dilute aPrV vaccine and further study is required to evaluate the solution to improve the vaccination against PrV in swine and to explore detailed molecular mechanisms underlying.

## Figures and Tables

**Table 1:** Sequences of primers for quantitative RT-PCR of transcription factor.

Gene	Primer sequence
GAPDH	Forward: 5-TCG TCC GGT AGA CAA AAT GG-3 Reverse: 5-GAG GTC AAT GAA GGG GTC GT-3

GATA-3	Forward: 5-GAG GTG GTG TCT GCA TTC CAA-3 Reverse: 5-TTT CAC AGC ACT AGA GAC CCT GTTA-3

T-bet	Forward: 5-GTT CCC ATT CCT GTC CTTC-3 Reverse: 5-CCT TGT TGT TGG TGA GCTT-3

**Table 2:** Design of experiment A in Section 2.12.

Groups	No. of mice	Treatment	
		Left hind limb	Right hind limb
1	6	aP-S	GSLS-Se
2	6	aP-GSe	Saline
3	6	aP-GSe	/
4	6	aP-S	Saline
5	6	No immunization	

GSLS-Se: saline solution containing GSLS and Se; aP-S: aPrV vaccine diluted with saline; aP-GSe: aPrV vaccine diluted with saline containing GSLS-Se; “/”: without treatment.

**Table 3:** Design of experiment B in Section 2.12.

Groups	No. of mice	Treatment before vaccination	Vaccination
1	6	/	aP-S
2	6	Injected with saline for 3 days	aP-S
3	6	Injected with GSLS-Se for 3 days	aP-S
4	6	/	aP-GSe
5	6	No immunization

GSLS-Se: saline solution containing GSLS and Se; aP-S: aPrV vaccine diluted with saline; aP-GSe: aPrV vaccine diluted with saline containing GSLS-Se; “/”: without treatment.

## Data Availability

The data used to support the findings of this study are available from the corresponding author upon request.
